# Bright’s Disease

**Published:** 1891-05

**Authors:** J. H. Sargent


### BRIGHT'S DISEASE.

The name of this dread disease originates in the fact that it was first described by Dr. Bright, of London.

It has two forms—the acute and the chronic, both of which have sub-divisions. The acute form is caused by taking cold, checked perspiration, intemperance, and especially scarlet fever, and its symptoms are pain in the back, diminution of the urine, irritability of the bladder, fever, nausea, frequent vomiting, dryness of skin, loss of appetite, dropsy noticed first about the eyes in the morning, followed by dropsy of lower limbs and general dropsical manifestations. The "Bright's disease" which is the subject of so much popular conversation at present is of a chronic nature and includes many diseased conditions of the kidneys, so that the name "Bright's diseases" would be a more appropriate appellation ; this, in fact, was the title Dr. Bright first gave these disorders.

It is well to have a general knowledge of some of the premonitory symptoms, for the reason that some forms of Bright's disease are unattended with pain at their inception, and the appetite and digestion in many cases are absolutely unaffected. Often there is no perceptible disturbance of the general nutrition for a long period after the disease commences, the patient preserves his strength and power in many instances for months after disease is steadily undermining his health and bringing him to a premature end.

These premonitory symptoms are severe attacks of headache of remarkably long duration, occurring often in middle life in an apparently healthy person ; sleeplessness and unrest, the sleep broken by troubled dreams ; disorders of vision, attended with repulsive smell of the breath ; occasional attacks of palpitation of the heart accompanied with vertigo, and a feeling of suffocation or want of breath ; nervous irritability, abnormal depression and moroseness ; an excessive quantity of urine secreted every twenty-four hours.

Occasional symptoms are signs of dyspepsia, headache, a marked tendency to perspire, pallor of the skin and a bloated appearance of the hands and particularly of the lower eyelids, and palpitation. Then are witnessed somewhat frequent attacks of acute stomach derangement, accompanied by severe retching, and commencing without any assignable cause, but often preceded or accompanied by a marked temporary decrease in the amount of urine secreted. The heart's action continues violent and anaemia advances. Dropsy is rarely prominent except in a mild form and near the end. The nervous system becomes affected, and not infrequently there are witnessed violent convulsions. The eyesight becomes impaired.

Finally the patient dies, usually from secondary mischief, such as pleurisy, erysipelas, bronchitis or pneumonia. But it should be always understood that a person may have one, or many, or all of the above named symptoms and yet not have any of Bright's diseases. What we wish to indicate here is, that a person noticing these symptoms should proceed at once to visit some physician and thus ascertain what is the real trouble. It is the practice of patent medicine vendors to fill their advertisements and almanacs with detailed descriptions of these symptoms for the purpose of frightening people into the belief that they have Bright's disease, with the expectation that a sale of their "infallible remedy" will be the natural result. To all this rot and rubbish, sensible people are not to listen. If you have any alarming indications of approaching physical danger, go to the tried and trusted old doctor and get his mature and skilled opinion on the matter.—*J. H. Sargent, M.D., in the Healthy Home.*

